# New evidence for SPX2 in regulating the brain-pituitary reproductive axis of half-smooth tongue sole (*Cynoglossus semilaevis*)

**DOI:** 10.3389/fendo.2022.984797

**Published:** 2022-08-01

**Authors:** Bin Wang, Kaijie Wang, Zhenfang Tian, Aijun Cui, Xin Liu, Zhixin Jin, Xuezhou Liu, Yan Jiang, Yongjiang Xu

**Affiliations:** ^1^ Key Laboratory of Sustainable Development of Marine Fisheries, Ministry of Agriculture and Rural Affairs, Yellow Sea Fisheries Research Institute, Chinese Academy of Fishery Sciences, Qingdao, China; ^2^ Joint Laboratory for Deep Blue Fishery Engineering, Pilot National Laboratory for Marine Science and Technology (Qingdao), Qingdao, China; ^3^ National Engineering Research Center For Marine Aquaculture, Zhejiang Ocean University, Zhoushan, China; ^4^ College of Fisheries, Tianjin Agricultural University, Tianjin, China; ^5^ College of Fisheries and Life Science , Dalian Ocean University, Dalian, China

**Keywords:** spexin, GnIH, GnRH, kisspeptin, gonadotropin, reproduction

## Abstract

Spexin (SPX) is an evolutionarily conserved neuropeptide, which was first identified in human proteome by data mining. Two orthologs (SPX1 and SPX2) are present in some non-mammalian species, including teleosts. It has been demonstrated that SPX1 is involved in reproduction and food intake, whereas the functional role of SPX2 is still absent in any vertebrate. The aim of the current study was to evaluate the actions of intraperitoneal injection of endogenous SPX2 peptide on the expression levels of some key reproductive genes of the brain-pituitary axis in half-smooth tongue sole. Our data showed an inhibitory action of SPX2 on brain *gnih*, *spx1*, *tac3* and pituitary *gthα*, *lhβ* mRNA levels. However, SPX2 had no significant effect on brain *gnihr*, *gnrh2*, *gnrh3*, *kiss2*, *kiss2r*, *spx2* expression or pituitary *gh* expression. On the other hand, SPX2 induced an increase in pituitary *fshβ* expression. Taken together, our results provide initial evidence for the involvement of SPX2 in the regulation of reproduction in vertebrates, which is in accordance with previous studies on SPX1.

## Introduction

Spexin (SPX), also termed neuropeptide Q (NPQ) or C12ORF39, is a novel hypothalamic neuropeptide that was first identified by bioinformatics approach (1, 2) , and subsequently its orthologs have been found from fish to mammals (3–5). The SPX mature peptide is a tetradecapeptide that is flanked by two dibasic protein cleavage sites (RR and GRR), and its amino acid sequence is highly conserved in various vertebrates, with only one amino acid substitution at position 13 between tetrapods and teleosts (3–5). Consistent with its widespread distribution in different tissues of teleosts and other vertebrates, SPX participates in a variety of physiological functions, such as glucose homeostasis, lipid metabolism, feeding, digestion, reproduction, among others (6–10). Preliminary evidence in teleosts and other vertebrates have indicated that SPX binds to the membrane galanin receptor 2 (Galr2) and Galr3, but not Galr1, to exert its functions (11–13).

Based on data acquisition and comparative synteny analysis, a novel SPX form, namely SPX2, has been identified in a few non-mammalian species, such as chicken, anole lizard, *Xenopus tropicalis*, zebrafish, medaka, and coelacanth (11). However, SPX2 is absent in mammals and the initial SPX is designated as SPX1 now (11). Interestingly, Nile tilapia and other cichlid fish species have two SPX1 paralogs (SPX1a and SPX1b) but have no SPX2 (12). In teleosts, the physiological functions of SPX are just emerging, and mainly focus on the control of reproduction and appetite (3–5). For instance, *in vivo* and *in vitro* administration of SPX1 suppress LH secretion in goldfish (14), and both LH and FSH plasma levels are significantly reduced after a single intraperitoneal injection of SPX1a or SPX1b in Nile tilapia (12). However, the reproductive capability is not impaired in SPX1 mutant zebrafish, suggesting that SPX1 is not essential for reproduction in this species (15).

SPX1 expression can be altered by nutritional status in several fish species (12, 16–22), and SPX1 has been shown to act as a satiety factor in goldfish (16) and zebrafish (15). Moreover, overexpression of SPX1 in the dorsal habenula reduces anxiety in zebrafish (23). On the other hand, no information is available regarding the biological role of SPX2 in any vertebrate, other than two studies on the SPX2-Galr2/3 interaction and detailed brain distribution of SPX2 in zebrafish (3–5). The serum response element-driven luciferase (SRE-luc) activity is significantly elevated by zebrafish SPX2 in HEK293 cells expressing zebrafish, *Xenopus*, and human Galr2 or Galr3, suggesting that SPX2 is an endogenous ligand for Galr2/3 (11). Recent data in zebrafish have revealed that SPX2 expression is restricted in the preoptic area of the hypothalamus by whole-mount *in situ* RNA hybridization, implying that SPX2 is implicated in reproduction and feeding control in this species (24). Accordingly, further investigation is urgently needed to clarify the potential role of SPX2 in vertebrates.

In all vertebrates, reproduction is mainly regulated by the brain-pituitary-gonadal (BPG) axis. A plethora of neuropeptides are involved in the control of reproduction, including gonadotropin-releasing hormone (GnRH), kisspeptin (Kiss), gonadotropin-inhibitory hormone (GnIH), neurokinin B (NKB), among others (25–35). Half-smooth tongue sole (*Cynoglossus semilaevis*) is an economically important marine flatfish that is widely cultured in China, and this species needs approximately 3 years of sexual maturation. In nature, the body length of mature females is twice larger and the body weight is over six times greater than those of mature males, exhibiting a sexual dimorphism of growth (36). Genes encoding these key factors have been cloned in half-smooth tongue sole, namely *gnrh2* (37), *gnrh3* (38), *kiss2* (39), Kiss2 receptor (*kiss2r*) (36), *gnih* (40), GnIH receptor (*gnihr*) (41), and *tac3* (42). Furthermore, growth hormone (*gh*) and three gonadotropin subunits (*gthα*, *lhβ*, and *fshβ*) are also available in this species (43, 44). Previous studies have indicated the existence of SPX1 and SPX2 in half-smooth tongue sole, and SPX1 exerts an action on the expression levels of brain and pituitary reproductive genes (20, 45). Herein, this study aimed to further clarify the possible role of SPX2 in the regulation of reproduction in this flatfish species.

## Materials and methods

### Animals

Approximately 2-year-old immature female tongue sole (body weight (BW), total length (TL) and gonadosomatic index (GSI) of 772.61 ± 25.69 g, 49.97 ± 0.51 cm and 2.66 ± 0.25%, respectively) were purchased from Haiyang Yellow Sea Aquatic Product Co., Ltd. (Haiyang, China), and maintained in an indoor concrete tank with recirculating seawater (water temperature 21–23°C and dissolved oxygen > 6 mg/L). Fish specimens were acclimatized for one week under a cyclical light–dark photoperiod (12 h: 12 h) and fed to satiation twice daily with commercial dry pellets. The animal study protocol was approved by the Animal Care and Use Committee of Yellow Sea Fisheries Research Institute, Chinese Academy of Fishery Sciences (ID Number: YSFRI-2021025).

### Peptide synthesis

The tongue sole SPX2 mature peptide (45) with amidation at the C-terminus (LNIHWGPQSMMYLKGKY-NH2) was synthesized by ChinaPeptides Co., Ltd. (Shanghai, China) with a purity of 95%, as determined by HPLC. The SPX2 peptide was dissolved in phosphate-buffered saline (PBS) just before the intraperitoneal injection experiments.

### *In vivo* effects of SPX2 on the brain-pituitary reproductive axis

SPX2 *in vivo* treatment experiments were generally performed as the previous study on tongue sole SPX1 (20). After acclimatization for one week as mentioned above, the fish were divided into three groups, anesthetized with MS222 (Sigma, 200 mg/L), weighed, and injected intraperitoneally with SPX2 peptide at two doses (100 ng/g BW and 1000 ng/g BW) or PBS alone (n = 8 fish/group). The injection volume of each dosage varied depending on the body weight of each fish. The whole brain and pituitary tissues were collected 6 h after the injection, frozen in liquid nitrogen, and stored at −80°C until use.

### RNA extraction, reverse transcription, and real-time quantitative PCR

All experiments were performed as described previously (46). Total RNA was isolated using the RNAiso Plus reagent (Takara), and 1 μg of total RNA was used as a template for the first-strand cDNA synthesis using the PrimeScript™ RT reagent Kit with gDNA Eraser (Takara). Real-time quantitative PCR analysis was performed on the LightCycler^®^ 96 PCR Instrument (Roche) using TB Green^®^ *Premix Ex Taq™* II (Takara) and the specific primers (**Table 1**). The thermal cycling profiles were as follows: 95°C for 30 s, and 40 cycles of 95°C for 5 s, 60°C for 20 s, and 72°C for 10 s. Melting curve analysis was performed in order to confirm the specificity of each product. *18s* ribosomal RNA was used as the internal reference for data normalization. The relative expression levels of each gene were normalized against those of the housekeeping gene and calculated by the comparative Ct method.

**Table 1:** List of primers for real-time quantitative PCR.

Name	Primer sequence (5'-3')	Amplicon size (bp)	GenBank accession NO.
*gnih-F*	GGAAATCAGCCTACAGTGACAAAA	120	KU612223
*gnih-R*	GCCTCTCCAAGTCCAAACTCC		
*gnihr-F*	GCTTTTCATGTTGTCCTGGTTG	147	KX839491
*gnihr-R*	GGGTTGATGCTTGAGTTGGAG		
*gnrh2 -F*	GGAATCTGAACTGGAGAACTGCT	121	KX090947
*gnrh2 -R*	TGGCTGCTCACAACTTTATCAC		
*gnrh3 -F*	AGGCAGCAGAGTGATCGTG	92	JQ028869
*gnrh3 -R*	CACCTGGTAGCCATCCATAAGAC		
*kiss2-F*	GGCAACTGCTGTGCAACGA	133	KX090946
*kiss2-R*	AAGACAGAAAGCGGGGAGAAC		
*kiss2r-F*	AGTTGTGATCGTCCTCCTCTTTG	92	KX685668
*kiss2r-R*	AGTTGGGTTGGTATTTGGGATG		
*spx1 -F*	GCTCCTAAGGGTTCGTTCCA	185	MG775238
*spx1 -R*	AGTATGGTGGCTGCCTGGTC		
*spx2 -F*	TCGTTAATCGCCTCCCTGTT	137	MH782165
*spx2 -R*	AGTGGTGCCTTGTTGTTCTCCT		
*tac3-F*	TCTGGTCCTCGTCGTCAAAC	175	MK336423
*tac3-F*	CGTGTTCCTTCTGCCCATC		
*gh-F*	TTATAGACCAGCGGCGTTTC	179	HQ334196
*gh-R*	ATGCTTGTTGTCGGGGATG		
*gthα-F*	TTCCCCACTCCTCTAACGACA	116	JQ364953
*gthα-R*	ACCACAATACCAGCCACCACTAC		
*lhβ-F*	TCCACCTGACACTAACGCTG	191	JQ277934
*lhβ-R*	GTTTGGTTCCTTTGTTCTGC		
*fshβ-F*	TGATGGGTGTCCAGAGGAAG	*95*	JQ277933
*fshβ-R*	CAACAAACCGTCCACAGTCC		
*18s-F*	GGTCTGTGATGCCCTTAGATGTC	107	GQ426786
*18s-R*	AGTGGGGTTCAGCGGGTTAC		

### Statistical analysis

Data were analyzed by one-way ANOVA followed by Duncan’s multiple comparison test using SPSS17.0, and are presented as mean ± SEM. Differences were considered statistically significant at *p* < 0.05.

## Results

### Effects of SPX2 peptide on the brain gene expression

**Figure 1:**
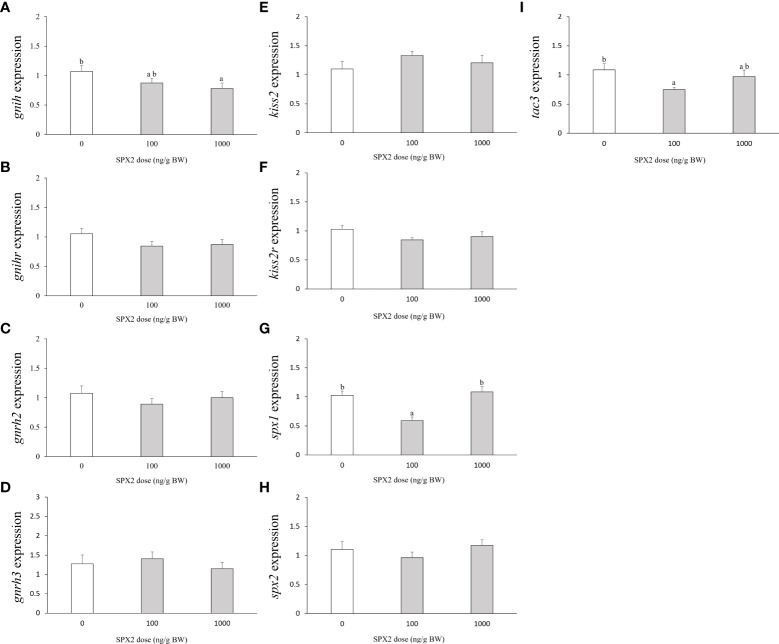
Effects of intraperitoneal injection of SXP2 peptide on brain *gnih* **(A)**, *gnihr* **(B)**, *gnrh2* **(C)**, *gnrh3* **(D)**, *kiss2* **(E)**, *kiss2r* **(F)**, *spx1* **(G)**, *spx2* **(H)** and *tac3* **(I)** transcript levels in tongue sole. Data are presented as mean ± SEM (n = 7-8). Different letters indicate significant differences between groups (*p* < 0.05).

First of all, we studied the *in vivo* effects of tongue sole SPX2 peptide on the expression levels of *gnih* and its cognate receptor *gnihr* genes in the brain (**Figures 1A, B**). Intraperitoneal injection of SPX2 at 1000 ng/g BW significantly inhibited *gnih* mRNA levels when compared to the control group (**Figure 1A**). However, no apparent variation in *gnihr* expression was noticed after administration of SPX2 at any of the two doses (**Figure 1B**).

Second, to investigate whether the GnRH system is a target of SPX2 action, brain expression levels of *gnrh2* and *gnrh3* were examined after treatment with SPX2 peptide (**Figures 1C, D**). Neither *gnrh2* nor *gnrh3* mRNA transcripts were altered by administration of SPX2 at the two doses tested (**Figures 1C, D**).

Third, we further evaluated the central action of SPX2 on the kisspeptin system (*kiss2* and its cognate receptor *kiss2r*). Similarly, SPX2 had no significant effects on brain *kiss2* and *kiss2r* mRNA levels compared to the control group (**Figures 1E, F**).

Fourth, to analyze the autocrine regulation of the spexin system, we examined the brain expression levels of *spx1* and *spx2* after administration of SPX2 peptide (**Figures 1G, H**). Only the fish treated with SPX2 at 100 ng/g BW showed an evident reduction in *spx1* mRNA levels (**Figure 1G**). However, *spx2* mRNA levels were not modified by the SPX2 peptide at the two doses tested (**Figure 1H**).

Finally, we detected the effects of SPX2 on the expression levels of *tac3* expressed in the brain (**Figure 1I**). Only SPX2 at the dose of 100 ng/g BW exerted an inhibitory action on *tac3* expression levels (**Figure 1I**).

### Effects of SPX2 peptide on the pituitary gene expression

**Figure 2:**
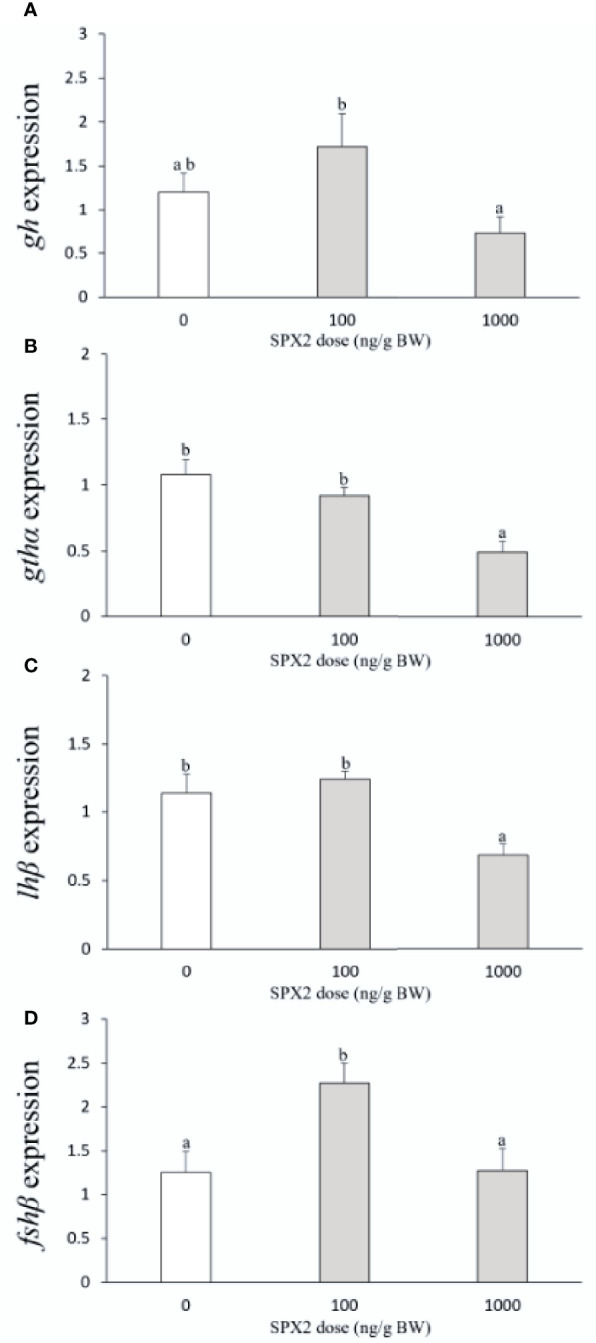
Effects of intraperitoneal injection of SXP2 peptide on pituitary *gh* **(A)**, *gthα* **(B)**, *lhβ* **(C)** and *fshβ* **(D)** transcript levels in tongue sole. Data are presented as mean ± SEM (n = 6-8). Different letters indicate significant differences between groups (*p* < 0.05).

As shown in **Figure 2A**, *gh* mRNA levels were not significantly altered by SPX2 at the two doses tested when compared to the control group. For *gthα* and *lhβ*, a significant suppression in their mRNA levels was observed by SPX2 at 1000 ng/g BW (**Figures 2B, C**). In contrast, SPX2 markedly stimulated *fshβ* mRNA levels with the lower dose of 100 ng/g BW when compared to the control group (**Figure 2D**).

## Discussion

SPX, which was first discovered by bioinformatics tools, is a newly described neuropeptide with pleiotropic functions in mammals (3, 4). Two SPX orthologs (SPX1 and SPX2) have been reported in some non-mammalian species, while the physiological functions of SPX are still largely unknown and remain to be investigated in this group of vertebrates. In bony fish, SPX1 exerts an inhibitory effect on reproduction (12, 14) and food intake (15, 16). However, no information exists about the potential biological functions of SPX2 in any vertebrate (3, 4). In the current study, therefore, half-smooth tongue sole was used as a model to investigate the *in vivo* actions of SPX2 on expression levels of reproductive genes in the brain-pituitary axis.

There is compelling evidence supporting that GnIH plays a critical role in the regulation of reproduction by acting at three levels of the BPG axis from fish to humans *via* its cognate receptor GnIHR (25, 27, 47). Our previous studies also have revealed that GnIH1 and GnIH2 peptides encoded by the same precursor exert a direct action on mRNA levels of pituitary hormones through the PKA and PKC signaling pathways in half-smooth tongue sole (41, 46). Results obtained in the present study indicated that intraperitoneal injection of SPX2 reduced *gnih* mRNA levels, without any effect on *gnihr* expression. In contrast, administration of SPX1 provoked an increase of *gnih* mRNA levels in immature females of the same species (20). Overall, these data suggest that SPX2 is implicated in the control of reproduction, while SPX1 and SPX2 may have different biological roles in half-smooth tongue sole. It is worth mentioning that Galr2 is an alternative endogenous receptor for SPX in zebrafish and Nile tilapia (11, 12). However, the morphological relationship between SPX and GnIH neurons is very limited in fish, thus much more studies need to be done to unveil whether Galr2 exists in GnIH neurons of half-smooth tongue sole and other species.

GnRH has been well demonstrated to be a master stimulator of the reproductive axis in vertebrates, and two or three distinct GnRH isoforms (GnRH1, GnRH2, and GnRH3) exist in all teleosts investigated so far. The brain distribution and physiological functions of these three GnRH variants are quite different. In a teleost species possessing all three GnRH types, GnRH1 is the main hypophysiotropic hormone regulating the BPG axis. However, GnRH3 takes over the role of GnRH1 in other teleost species that have GnRH2 and GnRH3 only (28, 29). One of the other key hypothalamic neuropeptides established in the control of reproduction is kisspeptin, which can exhibit potent action on pituitary directly or on GnRH neurons indirectly to regulate LH and FSH synthesis and secretion (30, 32, 33). In this study, none of *gnrh2*, *gnrh3*, *kiss2* or *kiss2r* expression levels were altered by SPX2 injection, indicating that the GnRH and kisspeptin systems may be not the central targets of SPX2 action on reproduction. Similarly, there is no significant effect of SPX1 on *gnrh1* and *gnrh2* mRNA levels in orange-spotted grouper (17) and immature females of half-smooth tongue sole (20), respectively. However, *gnrh3* expression is evidently elevated after SPX1 administration in the latter (20). Therefore, SPX1 and SPX2 might regulate different aspects of fish physiology.

In the present study, we evaluated the effects of SPX2 on the autocrine and paracrine regulation of spexin system. Peripheral injection of SPX2 suppressed *spx1* expression, without any effect on *spx2* mRNA levels. It has been demonstrated that SPX1 is involved in feeding, reproduction, and other functions in fish (3, 4), and these data indicate that SPX2 may participate in these physiological processes *via* SPX1 indirectly. Whether SPX1 has any effect on *spx2* expression is still unknown, which warrants further studies in various vertebrates. On the other hand, NKB encoded by the *tac3* gene has emerged as a key regulator of reproduction in mammals (34) and several teleost species, including zebrafish (48, 49), Nile tilapia (50), goldfish (51, 52), striped bass (53), European eel (54), and half-smooth tongue sole (42). Results obtained in this study indicated that SPX2 can reduce brain *tac3* mRNA levels, suggesting the regulation of reproduction by SPX2 *via* NKB indirectly.

In addition to its effects on brain functions, SPX can also modulate the synthesis and release of pituitary hormones. On one hand, both *gthα* and *lhβ* expression were down-regulated after intraperitoneal injection of SPX2, whereas *fshβ* mRNA levels were up-regulated in half-smooth tongue sole. On the other hand, SPX1 suppressed the expression levels of *gthα* and *fshβ*, without affecting *lhβ* expression in immature females of the same species (20). Neither *lhβ* nor *fshβ* transcripts were modified after SPX1 treatment in orange-spotted grouper (17). For hormone secretion, an inhibitory action of SPX1 on the plasma LH level was observed in goldfish and Nile tilapia (12, 14) along with a reduction of plasma FSH level in the latter. Interestingly, SPX1 evoked a decrease in the serum LH level, but an increase in the serum FSH level in mature female rats (55). Of note, SPX2 had no effect on *gh* expression in this study. However, SPX1 reduced *gh* mRNA levels in orange-spotted grouper and half-smooth tongue sole immature females (17, 20). It is worth mentioning that sexually immature female specimens were used in this study, and sexual maturity could be a contributing factor influencing the obtained results. Accordingly, further studies in sexually mature females during the seasonal reproductive cycle will contribute to a more complete picture of these two SPX peptides in this species. Taken together, SPX2 can modulate the reproduction of half-smooth tongue sole through actions on the expression of the components of brain-pituitary reproductive axis, and SPX1 and SPX2 seem to have divergent roles in the same species.

Despite its functional significance, the molecular mechanisms of SPX actions are incipient in vertebrates. A ligand-receptor interaction study has revealed that both SPX1 and SPX2 could increase SRE-luc activity in HEK293 cells expressing zebrafish Galr2a and Galr2b (11). Both SRE-luc and cAMP-response element luciferase (CRE-luc) activities are significantly elevated after SPX1a or SPX1b treatment in COS-7 cells expressing tilapia Galr2b (12). These data indicate that SPXs are a functional agonist for Galr2, and both PKC and PKA pathways mediate SPX functions. It is worth mentioning that clarifying the intricate web of intracellular pathways in response to SPX and its interaction with GnRH (28, 56), GnIH (57, 58), and kisspeptin (32, 35), is a promising area for future research not only in fish but also in other vertebrates.

## Conclusions

In summary, this study provides preliminary evidence for the involvement of SPX2 in the regulation of reproduction in vertebrates by acting at the brain and pituitary levels. Combined with previous studies on SPX1, it appears that some functional divergences exist between SPX1 and SPX2 peptides, perhaps due to the differences in their structures and binding affinity to their cognate receptors. Further studies on the molecular mechanisms involved in SPX actions on the target cells would contribute to the knowledge of the functional significance and divergence of this emerging neuropeptide in vertebrate species.

## Data availability statement

The original contributions presented in the study are included in the article/supplementary material. Further inquiries can be directed to the corresponding author.

## Ethics statement

The animal study was reviewed and approved by the Animal Care and Use Committee of Yellow Sea Fisheries Research Institute, Chinese Academy of Fishery Sciences.

## Author contributions

BW: conceptualization, validation, investigation, writing—original draft preparation, funding acquisition; KW: validation, investigation; ZT: investigation; AC: investigation; XiL: investigation; ZJ: investigation; XuL: resources, writing—review and editing; YJ: formal analysis; YX: validation, writing—review and editing, supervision, funding acquisition.

## Funding

This research was funded by the National Natural Science Foundation of China (32072949, 32072993), Central Public-interest Scientific Institution Basal Research Fund, YSFRI, CAFS (NO. 20603022022018), Central Public-interest Scientific Institution Basal Research Fund, CAFS (NO. TD202047), and China Agriculture Research System of MOF and MARA (CARS-47).

## Conflict of interest

The authors declare that the research was conducted in the absence of any commercial or financial relationships that could be construed as a potential conflict of interest.

## Publisher’s note

All claims expressed in this article are solely those of the authors and do not necessarily represent those of their affiliated organizations, or those of the publisher, the editors and the reviewers. Any product that may be evaluated in this article, or claim that may be made by its manufacturer, is not guaranteed or endorsed by the publisher.

## References

[1] Mirabeau O Perlas E Severini C Audero E Gascuel O Possenti R. Identification of novel peptide hormones in the human proteome by hidden Markov model screening. Genome Res (2007) 17(3):320–7. doi: 10.1101/gr.5755407 PMC1800923 17284679

[2] Sonmez K Zaveri NT Kerman IA Burke S Neal CR Xie X. Evolutionary sequence modeling for discovery of peptide hormones. PloS Comput Biol (2009) 5(1):e1000258. doi: 10.1371/journal.pcbi.1000258 19132080 PMC2603333

[3] Lim CH Lee MYM Soga T Parhar I. Evolution of structural and functional diversity of spexin in mammalian and non-mammalian vertebrate species. Front Endocrinol (Lausanne) (2019) 10:379. doi: 10.3389/fendo.2019.00379 31275244 PMC6593056

[4] Ma A Bai J He M Wong AOL. Spexin as a neuroendocrine signal with emerging functions. Gen Comp Endocrinol (2018) 265:90–6. doi: 10.1016/j.ygcen.2018.01.015 29355530

[5] Mohd Zahir I Ogawa S Dominic NA Soga T Parhar IS. Spexin and galanin in metabolic functions and social behaviors with a focus on non-mammalian vertebrates. Front Endocrinol (Lausanne) (2022) 13:882772. doi: 10.3389/fendo.2022.882772 35692389 PMC9174643

[6] Mills EG Izzi-Engbeaya C Abbara A Comninos AN Dhillo WS. Functions of galanin, spexin and kisspeptin in metabolism, mood and behaviour. Nat Rev Endocrinol (2021) 17(2):97–113. doi: 10.1038/s41574-020-00438-1 33273729

[7] Behrooz M Vaghef-Mehrabany E Maleki V Pourmoradian S Fathifar Z Ostadrahimi A. Spexin status in relation to obesity and its related comorbidities: a systematic review. J Diabetes Metab Disord (2020) 19(2):1943–57. doi: 10.1007/s40200-020-00636-8 PMC7843752 33520870

[8] Kolodziejski PA Pruszynska-Oszmalek E Wojciechowicz T Sassek M Leciejewska N Jasaszwili M. The role of peptide hormones discovered in the 21st century in the regulation of adipose tissue functions. Genes (Basel) (2021) 12(5):756. doi: 10.3390/genes12050756 34067710 PMC8155905

[9] Tran A He W Chen JTC Belsham DD. Spexin: Its role, regulation, and therapeutic potential in the hypothalamus. Pharmacol Ther (2021) p:108033. doi: 10.1016/j.pharmthera.2021.108033 34763011

[10] Lv SY Zhou YC Zhang XM Chen WD Wang YD. Emerging roles of NPQ/Spexin in physiology and pathology. Front Pharmacol (2019) 10:457. doi: 10.3389/fphar.2019.00457 31133851 PMC6514225

[11] Kim DK Yun S Son GH Hwang JI Park CR Kim JI. Coevolution of the spexin/galanin/kisspeptin family: Spexin activates galanin receptor type II and III. Endocrinology (2014) 155(5):1864–73. doi: 10.1210/en.2013-2106 24517231

[12] Cohen Y Hausken K Bonfil Y Gutnick M Levavi-Sivan B. Spexin and a novel cichlid-specific spexin paralog both inhibit FSH and LH through a specific galanin receptor (Galr2b) in tilapia. Front Endocrinol (Lausanne) (2020) 11:71. doi: 10.3389/fendo.2020.00071 32153508 PMC7044129

[13] Lin CY Zhang M Huang T Yang LL Fu HB Zhao L. Spexin enhances bowel movement through activating l-type voltage-dependent calcium channel *via* galanin receptor 2 in mice. Sci Rep (2015) 5:12095. doi: 10.1038/srep12095 26160593 PMC4498193

[14] Liu Y Li S Qi X Zhou W Liu X Lin H. A novel neuropeptide in suppressing luteinizing hormone release in goldfish, carassius auratus. Mol Cell Endocrinol (2013) 374(1-2):65–72. doi: 10.1016/j.mce.2013.04.008 23623870

[15] Zheng B Li S Liu Y Li Y Chen H Tang H. Spexin suppress food intake in zebrafish: Evidence from gene knockout study. Sci Rep (2017) 7(1):14643. doi: 10.1038/s41598-017-15138-6 29116147 PMC5677112

[16] Wong MK Sze KH Chen T Cho CK Law HC Chu IK. Goldfish spexin: solution structure and novel function as a satiety factor in feeding control. Am J Physiol Endocrinol Metab (2013) 305(3):E348–66. doi: 10.1152/ajpendo.00141.2013 23715729

[17] Li S Liu Q Xiao L Chen H Li G Zhang Y. Molecular cloning and functional characterization of spexin in orange-spotted grouper (*Epinephelus coioides*). Comp Biochem Physiol B Biochem Mol Biol (2016) 196-197:85–91. doi: 10.1016/j.cbpb.2016.02.009 26944307

[18] Wu H Lin F Chen H Liu J Gao Y Zhang X. Ya-fish (*Schizothorax prenanti*) spexin: identification, tissue distribution and mRNA expression responses to periprandial and fasting. Fish Physiol Biochem (2016) 42(1):39–49. doi: 10.1007/s10695-015-0115-0 26311351

[19] Ma A He M Bai J Wong MK Ko WK Wong AO. Dual role of insulin in spexin regulation: Functional link between food intake and spexin expression in a fish model. Endocrinology (2017) 158(3):560–77. doi: 10.1210/en.2016-1534 28359089

[20] Wang S Wang B Chen S. Spexin in the half-smooth tongue sole (*Cynoglossus semilaevis*): molecular cloning, expression profiles, and physiological effects. Fish Physiol Biochem (2018) 44(3):829–39. doi: 10.1007/s10695-018-0472-6 29404821

[21] Deng SP Chen HP Zhai Y Jia LY Liu JY Wang M. Molecular cloning, characterization and expression analysis of spexin in spotted scat (*Scatophagus argus*). Gen Comp Endocrinol (2018) 266:60–6. doi: 10.1016/j.ygcen.2018.04.018 29753927

[22] Tian Z Xu S Wang M Li Y Chen H Tang N. Identification, tissue distribution, periprandial expression, and anorexigenic effect of spexin in Siberian sturgeon, acipenser baeri. Fish Physiol Biochem (2020) 46(6):2073–84. doi: 10.1007/s10695-020-00856-y 32794103

[23] Jeong I Kim E Seong JY Park HC. Overexpression of spexin 1 in the dorsal habenula reduces anxiety in zebrafish. Front Neural Circuits (2019) 13:53. doi: 10.3389/fncir.2019.00053 31474838 PMC6702259

[24] Kim E Jeong I Chung AY Kim S Kwon SH Seong JY. Distribution and neuronal circuit of spexin 1/2 neurons in the zebrafish CNS. Sci Rep (2019) 9(1):5025. doi: 10.1038/s41598-019-41431-7 30903017 PMC6430828

[25] Trudeau VL. Neuroendocrine control of reproduction in teleost fish: Concepts and controversies. Annu Rev Anim Biosci (2022) 10:18.1–18.24. doi: 10.1146/annurev-animal-020420-042015 34788545

[26] Zohar Y Zmora N Trudeau VL Munoz-Cueto JA Golan M. A half century of fish gonadotropin-releasing hormones: Breaking paradigms. J Neuroendocrinol (2021) 34:e13069. doi: 10.1111/jne.13069 34913529

[27] Munoz-Cueto JA Paullada-Salmeron JA Aliaga-Guerrero M Cowan ME Parhar IS Ubuka T. A journey through the gonadotropin-inhibitory hormone system of fish. Front Endocrinol (Lausanne) (2017) 8:285. doi: 10.3389/fendo.2017.00285 29163357 PMC5670112

[28] Munoz-Cueto JA Zmora N Paullada-Salmeron JA Marvel M Mananos E Zohar Y. The gonadotropin-releasing hormones: Lessons from fish. Gen Comp Endocrinol (2020) 291:113422. doi: 10.1016/j.ygcen.2020.113422 32032603

[29] Duan C Allard J. Gonadotropin-releasing hormone neuron development in vertebrates. Gen Comp Endocrinol (2020) 292:113465. doi: 10.1016/j.ygcen.2020.113465 32184073

[30] Somoza GM Mechaly AS Trudeau VL. Kisspeptin and GnRH interactions in the reproductive brain of teleosts. Gen Comp Endocrinol (2020) 298:113568. doi: 10.1016/j.ygcen.2020.113568 32710898

[31] Di Yorio MP Munoz-Cueto JA Paullada-Salmeron JA Somoza GM Tsutsui K Vissio PG. The gonadotropin-inhibitory hormone: What we know and what we still have to learn from fish. Front Endocrinol (Lausanne) (2019) 10:78. doi: 10.3389/fendo.2019.00078 30837949 PMC6389629

[32] Ohga H Selvaraj S Matsuyama M. The roles of kisspeptin system in the reproductive physiology of fish with special reference to chub mackerel studies as main axis. Front Endocrinol (Lausanne) (2018) 9:147. doi: 10.3389/fendo.2018.00147 29670580 PMC5894438

[33] Sivalingam M Ogawa S Trudeau VL Parhar IS. Conserved functions of hypothalamic kisspeptin in vertebrates. Gen Comp Endocrinol (2022) 317:113973. doi: 10.1016/j.ygcen.2021.113973 34971635

[34] Hu G Lin C He M Wong AO. Neurokinin b and reproductive functions: "KNDy neuron" model in mammals and the emerging story in fish. Gen Comp Endocrinol (2014) 208:94–108. doi: 10.1016/j.ygcen.2014.08.009 25172151

[35] Wang B Mechaly AS Somoza GM. Overview and new insights into the diversity, evolution, role, and regulation of kisspeptins and their receptors in teleost fish. Front Endocrinol (Lausanne) (2022) 13:862614. doi: 10.3389/fendo.2022.862614 35392133 PMC8982144

[36] Wang B Liu Q Liu X Xu Y Shi B. Molecular characterization of Kiss2 receptor and *in vitro* effects of Kiss2 on reproduction-related gene expression in the hypothalamus of half-smooth tongue sole (*Cynoglossus semilaevis*). Gen Comp Endocrinol (2017) 249:55–63. doi: 10.1016/j.ygcen.2017.04.006 28438528

[37] Wang B Liu X Liu Q Zhao M Xu Y Shi B. Molecular cloning, localization, and expression analysis of gnrh2 in different tissues of half-smooth tongue sole (*Cynoglossus semilaevis*) during ovarian maturation. Prog Fish Sci (2017) 38(1):63–72. doi: 10.11758/yykxjz.20160816002

[38] Zhou X Yi Q Zhong Q Li C Muhammad S Wang X. Molecular cloning, tissue distribution, and ontogeny of gonadotropin-releasing hormone III gene (GnRH-III) in half-smooth tongue sole (*Cynoglossus semilaevis*). Comp Biochem Physiol B Biochem Mol Biol (2012) 163(1):59–64. doi: 10.1016/j.cbpb.2012.04.010 22580269

[39] Wang B Liu Q Liu X Xu Y Song X Shi B. Molecular characterization of *kiss2* and differential regulation of reproduction-related genes by sex steroids in the hypothalamus of half-smooth tongue sole (*Cynoglossus semilaevis*). Comp Biochem Physiol A Mol Integr Physiol (2017) 213:46–55. doi: 10.1016/j.cbpa.2017.08.003 28822779

[40] Wang B Liu Q Liu X Xu Y Shi B. Molecular characterization and expression profiles of LPXRFa at the brain-pituitary-gonad axis of half-smooth tongue sole (*Cynoglossus semilaevis*) during ovarian maturation. Comp Biochem Physiol B Biochem Mol Biol (2018) 216:59–68. doi: 10.1016/j.cbpb.2017.11.016 29223873

[41] Wang B Yang G Liu Q Qin J Xu Y Li W. Characterization of LPXRFa receptor in the half-smooth tongue sole (*Cynoglossus semilaevis*): Molecular cloning, expression profiles, and differential activation of signaling pathways by LPXRFa peptides. Comp Biochem Physiol A Mol Integr Physiol (2018) 223:23–32. doi: 10.1016/j.cbpa.2018.05.008 29746909

[42] Wang B Cui A Zhang Y Xu Y Wang W Jiang Y. Neurokinin b in a flatfish species, the half-smooth tongue sole (*Cynoglossus semilaevis*), and its potential role in reproductive functions. Aquac Rep (2021) 20:100651. doi: 10.1016/j.aqrep.2021.100651

[43] Ji XS Liu HW Chen SL Jiang YL Tian YS. Growth differences and dimorphic expression of growth hormone (GH) in female and male *Cynoglossus semilaevis* after male sexual maturation. Mar Genomics (2011) 4(1):9–16. doi: 10.1016/j.margen.2010.11.002 21429460

[44] Shi B Liu X Xu Y Wang S. Molecular characterization of three gonadotropin subunits and their expression patterns during ovarian maturation in *Cynoglossus semilaevis* . Int J Mol Sci (2015) 16(2):2767–93. doi: 10.3390/ijms16022767 PMC4346864 25633101

[45] Wang B Cui A Tian J Zhang Y Jiang Y Xu Y. Characterization of a novel spexin gene (*spx2*) in the half-smooth tongue sole and regulation of its expression by nutritional status. Aquac Rep (2020) 18:100544. doi: 10.1016/j.aqrep.2020.100544

[46] Wang B Yang G Xu Y Zhang Y Liu X. In vitro effects of tongue sole LPXRFa and kisspeptin on relative abundance of pituitary hormone mRNA and inhibitory action of LPXRFa on kisspeptin activation in the PKC pathway. Anim Reprod Sci (2019) 203:1–9. doi: 10.1016/j.anireprosci.2019.01.009 30797596

[47] Tsutsui K Ubuka T. Gonadotropin-inhibitory hormone (GnIH): A new key neurohormone controlling reproductive physiology and behavior. Front Neuroendocrinol (2021) 61:100900. doi: 10.1016/j.yfrne.2021.100900 33450199

[48] Biran J Palevitch O Ben-Dor S Levavi-Sivan B. Neurokinin bs and neurokinin b receptors in zebrafish-potential role in controlling fish reproduction. Proc Natl Acad Sci U.S.A. (2012) 109(26):10269–74. doi: 10.1073/pnas.1119165109 PMC3387093 22689988

[49] Qi X Salem M Zhou W Sato-Shimizu M Ye G Smitz J. Neurokinin b exerts direct effects on the ovary to stimulate estradiol production. Endocrinology (2016) 157(9):3355–65. doi: 10.1210/en.2016-1354 27580802

[50] Biran J Golan M Mizrahi N Ogawa S Parhar IS Levavi-Sivan B. Direct regulation of gonadotropin release by neurokinin b in tilapia (*Oreochromis niloticus*). Endocrinology (2014) 155(12):4831–42. doi: 10.1210/en.2013-2114 25211586

[51] Qi X Zhou W Li S Liu Y Ye G Liu X. Goldfish neurokinin b: Cloning, tissue distribution, and potential role in regulating reproduction. Gen Comp Endocrinol (2015) 221:267–77. doi: 10.1016/j.ygcen.2014.10.017 25448255

[52] Liu Y Wang Q Wang X Meng Z Liu Y Li S. NKB/NK3 system negatively regulates the reproductive axis in sexually immature goldfish (*Carassius auratus*). Gen Comp Endocrinol (2019) 281:126–36. doi: 10.1016/j.ygcen.2019.05.020 31163181

[53] Zmora N Wong TT Stubblefield J Levavi-Sivan B Zohar Y. Neurokinin b regulates reproduction *via* inhibition of kisspeptin in a teleost, the striped bass. J Endocrinol (2017) 233(2):159–74. doi: 10.1530/JOE-16-0575 28330973

[54] Campo A Lafont AG Lefranc B Leprince J Tostivint H Kamech N. Tachykinin-3 genes and peptides characterized in a basal teleost, the European eel: Evolutionary perspective and pituitary role. Front Endocrinol (Lausanne) (2018) 9:304. doi: 10.3389/fendo.2018.00304 29942283 PMC6004781

[55] Golyszny M Obuchowicz E Zielinski M. Neuropeptides as regulators of the hypothalamus-pituitary-gonadal (HPG) axis activity and their putative roles in stress-induced fertility disorders. Neuropeptides (2022) 91:102216. doi: 10.1016/j.npep.2021.102216 34974357

[56] Chang JP Pemberton JG. Comparative aspects of GnRH-stimulated signal transduction in the vertebrate pituitary - contributions from teleost model systems. Mol Cell Endocrinol (2018) 463:142–67. doi: 10.1016/j.mce.2017.06.002 28587765

[57] Son YL Ubuka T Tsutsui K. Molecular mechanisms of gonadotropin-inhibitory hormone (GnIH) actions in target cells and regulation of GnIH expression. Front Endocrinol (Lausanne) (2019) 10:110. doi: 10.3389/fendo.2019.00110 30858828 PMC6397841

[58] Wang B Yang G Xu Y Li W Liu X. Recent studies of LPXRFa receptor signaling in fish and other vertebrates. Gen Comp Endocrinol (2019) 277:3–8. doi: 10.1016/j.ygcen.2018.11.011 30465768

